# CEP131 Abrogates CHK1 Inhibitor-Induced Replication Defects and Is Associated with Unfavorable Outcome in Neuroblastoma

**DOI:** 10.1155/2020/2752417

**Published:** 2020-09-15

**Authors:** Kiyohiro Ando, Verna Cázares-Ordoñez, Makoto Makishima, Atsushi Yokoyama, Yusuke Suenaga, Hiroki Nagase, Shinichi Kobayashi, Takehiko Kamijo, Tsugumichi Koshinaga, Satoshi Wada

**Affiliations:** ^1^Department of Clinical Diagnostic Oncology, Showa University Clinical Research Institute for Clinical Pharmacology and Therapeutics, 6-11-11 Kita-Karasuyama, Setagaya-ku, Tokyo 157-8577, Japan; ^2^Research Institute for Clinical Oncology, Saitama Cancer Center, 818 Komuro, Ina, Saitama, Japan; ^3^Chiba Cancer Center Research Institute, 666-2 Nitona, Chiba 260-8717, Japan; ^4^Division of Biochemistry, Department of Biomedical Sciences, Nihon University School of Medicine, 30-1 Oyaguchi-Kamicho, Itabashi-ku, Tokyo 173-8610, Japan; ^5^Showa University Clinical Research Institute for Clinical Pharmacology and Therapeutics, 6-11-11 Kita-Karasuyama, Setagaya-ku, Tokyo 157-8577, Japan; ^6^Department of Molecular Endocrinology, Tohoku University Graduate School of Medicine, 2-1 Seiryo-Machi, Aoba-ku, Sendai 980-8575, Japan; ^7^Department of Pediatric Surgery, Nihon University School of Medicine, 30-1 Oyaguchi-Kamicho, Itabashi-ku, Tokyo 173-8610, Japan

## Abstract

Checkpoint kinase 1 (CHK1) plays a key role in genome surveillance and integrity throughout the cell cycle. Selective inhibitors of CHK1 (CHK1i) are undergoing clinical evaluation for various human malignancies, including neuroblastoma. Recently, we reported that CHK1i, PF-477736, induced a p53-mediated DNA damage response. As a result, the cancer cells were able to repair DNA damage and became less sensitive to CHK1i. In this study, we discovered that PF-477736 increased expression of MDM2 oncogene along with CHK1i-induced replication defects in neuroblastoma NB-39-nu cells. A mass spectrometry analysis of protein binding to MDM2 in the presence of CHK1i identified the centrosome-associated family protein 131 (CEP131), which was correlated with unfavorable prognosis of neuroblastoma patients. We revealed that MDM2 was associated with CEP131 protein degradation, whereas overexpression of CEP131 accelerated neuroblastoma cell growth and exhibited resistance to CHK1i-induced replication defects. Thus, these findings may provide a future therapeutic strategy against centrosome-associated oncogenes involving CEP131 as a target in neuroblastoma.

## 1. Introduction

Checkpoint kinase 1 (CHK1) is primarily responsible for regulating replication origin firing, stabilizing stalled replication forks at the intra-S phase checkpoint, and preventing mitotic onset at the G_2_/M checkpoint through phosphorylation of CDC25 family members [1]. Considering the vulnerability of cancer cells encumbered with genomic instability, the blockade of CHK1 is not only synergistic with chemotherapy, but may also be fatal to the cell as a result of excessive DNA damage. A number of small-molecule inhibitors targeting CHK1 (CHK1i) have been developed for cancer therapy [2–4]. However, clinical benefit has not yet been sufficiently provided; therefore clarification of the mechanistic underpinning cellular response to CHK1i is needed for improving the design of future clinical trials.

Recently, we reported that CHK1i (PF-477736) induced DNA damage leading to p53 activation in NB-39-nu neuroblastoma cells, which are relatively less sensitive to CHK1i [5]. This was accompanied by elevated transcription of the downstream cyclin-dependent kinase inhibitor CDKN1A (p21) and MDM2, an ubiquitin ligase of p53. In the event of DNA damage, p21 causes G1 arrest and prevents the damaged cell from entering S-phase until DNA lesions are repaired. MDM2 is sequestered by p19Arf preventing negative-feedback regulation from p53 protein degradation, thus resulting in less sensitivity to CHK1i-induced cell death [6, 7]. In this study, we unexpectedly observed that MDM2 protein expression was increased earlier than that of the p53 response following CHK1i exposure, suggesting that there exists an unknown physiological role of MDM2 that is p53-independent. A protein interaction analysis identified centrosome-associated protein 131 (CEP131) as a novel MDM2 interacting protein.

CEP131 (azacytidine-inducible-1, AZI-1) was initially discovered in the murine spermatid and is an evolutionarily conserved centrosomal protein in humans [8, 9]. Further studies revealed that CEP131 is a component of centriolar satellites associated with cilia formation [10–12]. Notably, it has been reported that experimental depletion of CEP131 induces centriole amplification resulting in increased frequency of multipolar mitosis, chromosomal instability, and postmitotic DNA damage [13]. Therefore, in addition to ciliogenesis, CEP131 plays a role in genome integrity during normal proliferation. As such, several lines of evidence show that increased expression of CEP131 is correlated with higher histologic grades of breast cancer and poor overall survival in hepatocellular carcinoma and advanced Tumor-Node-Metastasis stage of non-small cell lung cancer [14–16]. However, the direct contribution of CEP131 to cancer proliferation has been still contentious. In this study, we revealed that increased expression of CEP131 confer tolerability against replicative stress on cells and may be associated with proliferation in unfavorable neuroblastomas.

## 2. Materials and Methods

### 2.1. Cell Culture and Treatments

The human NB cell lines NB-39-nu, SMS-SAN, NBLS, CHP134, SH-SY5Y and SK-N-AS, and SK-N-BE were purchased from the American Type Culture Collection (Manassas, VA, USA) and the RIKEN Bioresource Cell Bank, Tohoku University Cell Resource Center (Miyagi, Japan). Cells were cultured in RPMI 1640 medium supplemented with 10% heat-inactivated fetal bovine serum (FBS), 50 *μ*g/ml penicillin, and 50 *μ*g/ml streptomycin (Thermo Fisher Scientific, Waltham, MA, USA). 293FT cell line (Thermo Fisher Scientific) was maintained in DMEM medium with 10% FBS, 50 *μ*g/ml penicillin, 50 *μ*g/ml streptomycin, and 500 *μ*g/ml Geneticin (Thermo Fisher Scientific). Cells were incubated at 37°C in a 5% CO_2_ humidified atmosphere (MCO-175-PJ, PHC, Tokyo, Japan). PF-477736 (henceforth, CHK1i) was purchased from Sigma-Aldrich (St. Louis, MO, USA). Stock solutions were made in dimethyl sulfoxide (DMSO).

### 2.2. Cell Viability Assays

NB-39-nu cells, which were transduced with lentiviral-mediated expression of LUCZ or CEP131, were seeded in each well of a 96-well plate (20,000 cells/well) and subsequently treated with 1 *μ*M CHK1i or DMSO. Forty-eight hours after incubation, ten microliters of alamarBlue Cell Viability Reagent (Invitrogen, Carlsbad, CA, USA) was added to the each well for 1 h, and immunofluorescence was measured using a SpectraMax M5e plate reader (Molecular Devices, San Jose, CA, USA).

### 2.3. Detection of S Phase Nuclei

For detection of S phase nuclei, cells were seeded into a 35 mm glass bottom dish (Matsunami Glass, Osaka, Japan) and treated with 1 *μ*M CHK1i for 30 min, 1 h, and 2 h or without CHK1i. After incubation, cells were exposed to 5-ethynyl-2ʹ-deoxyuridine and then stained by using the Click-iT EdU Imaging Kit (Thermo Fisher Scientific) according to the manufacturer's instructions. The duration of EdU exposure to cells was described in the corresponding figure legends. Cells were then mounted with VECTASHIELD Mounting Medium containing 4′,6-diamidino-2-phenylindole (DAPI) (Vector Laboratories, Burlingame, CA, USA) overlaid with cover glass (As one, Osaka, Japan). The nuclei stained with DAPI and EdU-labeled S phase nuclei were visualized and photographed using an A1 confocal microscope (Nikon, Tokyo, Japan). One hundred nuclei were counted in each of the triplicate sets and the relative numbers of S phase nuclei were scored.

### 2.4. Quantitative Reverse-Transcription PCR (qPCR)

Seven neuroblastoma cell lines, NB-39-nu, SMS-SAN, NBLS, CHP134, SH-SY5Y, SK-N-AS, and SK-N-BE, were treated with 1 *μ*M and 5 *μ*M CHK1i for 24 h and without CHK1i. Total RNA was extracted from the cell lines by using RNeasy Mini Kit (Qiagen, Hilden, Germany) and then reverse-transcribed using Superscript IV VILO Master Mix (Invitrogen) according to the manufacturer's instructions. Complementary DNA was amplified by real-time PCR to quantify the expression of MDM2 and *ACTB* mRNA. MDM2 and *ACTB* TaqMan probes (Hs01066930_m1 and Hs01060665_g1, resp.) were purchased from Applied Biosystems (Foster City, CA, USA). qPCR was performed using an Applied Biosystems StepOnePlus Real-Time PCR System with TaqMan Fast Universal PCR Master Mix according to the manufacturer's instructions. Relative quantification was determined by using the 2^−ΔΔCT^ method. Fold changes of transcript levels in each cell line with or without PF-477736 treatment were calculated by comparison to the no treatment control after normalization with *ACTB* expression. Data are presented as the mean ± SD of triplicates.

### 2.5. Mass Spectrometry

Anti-MDM2 immunoprecipitates were separated and visualized on NuPAGE 4–12% Bis-Tris gels (Invitrogen) followed by Coomassie staining with 0.12% Coomassie Brilliant Blue G-250 (Tokyo Chemical Industry Tokyo, Japan), 10% phosphoric acid, 10% ammonium sulfate, and 20% methanol. Protein bands were retrieved and analyzed by liquid chromatography-tandem mass spectrometry (LC-MS/MS). Digested peptides were cleaned using a Pierce C18Spin Column (Thermo Fisher Scientific) and analyzed on a LTQ-Orbitrap Velos spectrometer (Thermo Fisher Scientific). For protein identification, spectra were processed with Proteome Discoverer version 1.3 software (Thermo Fisher Scientific) using the Mascot algorithm and the Swiss-Prot human protein database. Peptide data were filtered using a Mascot significance threshold of less than 0.05 and Peptide Probability (FDR < 0.01).

### 2.6. Kaplan–Meier Survival Curves

Dataset of the overall survival of patients with primary neuroblastomas was obtained from “Tumor Neuroblastoma public-Versteeg-88-MAS5.0-u133p2” and analyzed using “R2: Genomics Analysis and Visualization Platform” according to the relative CEP131 expression levels. *P* value was determined by the log-rank test and mRNA expression was classified as high or low according to the median value.

### 2.7. Construction of Expression Vector and Stable Cell Line

The full-length cDNA for CEP131 was kindly provided by Dr. Lei Shi [14]. PCR amplification was performed using the cDNA template and the product was subcloned into pENTR/D-TOPO (Invitrogen) to generate an entry clone. The forward and reverse primers used for PCR amplification were 5′- CACCATGGAAGGCACCCGGGCCATCG-3′ and 5′- CTTGGTACTTGGCGTGGGCCTCCTG-3′. Generation of a stable cell line transduced by lentiviral-mediated expression of CEP131 was carried out using the ViraPower Lentiviral Gateway Expression Kit (Invitrogen) according to the manufacturer's instructions. Briefly, the cDNA was transferred into the pLenti6.2/V5-DEST vector to create the expression clone and transiently transfected with the ViraPower Packaging Mix into 293FT cells. Viral supernatants were collected and clarified by filtration 48 hours after transfection and then subsequently used to transduce NB-39-nu cells. Forty-eight hours after transduction, the cells underwent selection with blasticidin (3 *μ*g ml ^−1^, Thermo Fisher Scientific) for 14 days.

### 2.8. Immunoblotting and Co-Immunoprecipitation

Immunoblot analysis was performed as described previously [5]. See the Supplemental Materials (available here) and methods for further details. The primary antibodies used were as follows: monoclonal anti-p53 (DO-1, Santa Cruz Biotechnology; 1 : 500), monoclonal anti-*β*-actin (AC-74, Sigma; 1 : 4000), monoclonal anti-MDM2 (SMP14, Santa Cruz Biotechnology, Dallas, TX, USA; 1 : 200), monoclonal anti-p21 Waf1/Cip1 (12D1, Cell Signaling Technology, Danvers, MA, USA; 1 : 1000), monoclonal anti-V5 tag (Invitrogen; 1 : 1000), polyclonal anti-p-p53-Ser15, polyclonal anti-p-histone H2A.X-Ser139 (Cell Signaling Technology; 1 : 1000), or polyclonal anti-AZI1 (ab99379, Abcam, Cambridge, UK; 1 : 1000) antibody. For coimmunoprecipitation, whole-cell lysates were prepared in 1% NP-40 cell lysis buffer (Boston BioProducts) with Protease Inhibitor and phosphatase inhibitor, as described above. Whole-cell lysates (5 mg) were mixed with 20 *μ*l protein-A/G magnetic beads (Thermo Fisher Scientific) and 12.5 *μ*g (500 *μ*l final volume) mouse monoclonal anti-MDM2 antibody (SMP14, Santa Cruz Biotechnology) or normal mouse IgG (sc-2025, Santa Cruz Biotechnology) and incubated overnight at 4°C with gradual rotation. Beads were then washed three times with PBS/0.02% Triton X-100 and subjected to immunoblotting with the following primary antibodies: rabbit polyclonal anti-AZI1 antibody (ab99379, Abcam; 1 : 1000) and anti-MDM2 antibody (SMP14, Santa Cruz Biotechnology; 1 : 500) detected with HRP-conjugated anti-rabbit secondary antibodies (Cell Signaling Technology; 1 : 2500) or mouse TrueBlot HRP-conjugated secondary antibodies (eBioscience, Santa Clara, CA, USA; 1 : 1000).

### 2.9. Immunofluorescence Microscopy

Indirect immunofluorescence analysis was performed as described previously [5]. See the Supplemental Materials and Methods for further details. The primary antibodies used were as follows: monoclonal anti-V5 tag (Invitrogen; 1 : 100) or polyclonal anti-AZI1 (ab99379, Abcam; 1 : 100) antibody. Samples were visualized and photographed using an A1 confocal microscope (Nikon).

### 2.10. RNA Interference

Stealth RNAi, siRNA specific for MDM2 (MDM2HSS142909, 142910, and 142911) and a negative control sequence (medium GC-content) were obtained from Invitrogen. Cells were transiently transfected with 50 nM siRNA using Lipofectamine RNAiMAX (Invitrogen) reagent and were prepared 48 h after transfection for each experiment.

### 2.11. Cycloheximide Chase Assay

NB-39-nu cells were transfected with MDM2 siRNA or control siRNA. Forty-eight hours after incubation, cells were treated with 1 *μ*M PF-477736 for 30 min and then cycloheximide was added to the cells at final concentration 25 *μ*g/ml for 1 h, 2.5 h, or 4 h. After incubation, cells were harvested and lysed. Whole-cell extracts (100 *μ*g) were used for western blot analysis as described above.

### 2.12. Statistical Analysis

Statistical analysis was performed using Microsoft Excel (Microsoft, Redmond, WA) and Statcel 4 (OMS Publishing, Saitama, Japan). Statistical differences between two groups or four groups were analyzed using an unpaired Student's *t*-test or ANOVA test followed by Scheffé's F test, respectively. The results are presented as the mean ± standard deviation (SD) with a significance level of ^*∗*^*P* < 0.05 and ^*∗∗*^*P* < 0.001.

## 3. Results

### 3.1. Increased Levels of MDM2 Protein and Decreased DNA Replication Capacity in Response to CHK1i Exposure

**Figure 1:**
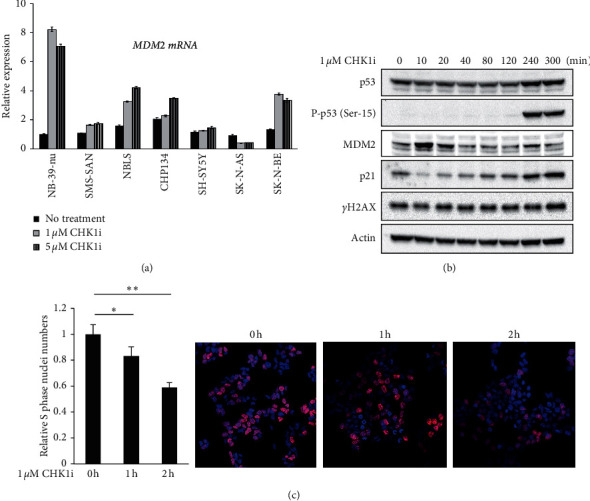
Increased expression of MDM2 and decreased DNA replication capacity were observed in response to Checkpoint kinase 1 inhibitor (CHK1i, PF-477736): (a) quantitative RT-PCR analysis of MDM2 mRNA levels in various neuroblastoma cell lines treated with 0, 1, and 5 *μ*M CHK1i for 24 h; (b) immunoblot analysis using anti-p53, anti-phosphorylated p53 at Ser^15^ (P-p53 ^Ser15^), anti-MDM2, anti-p21, or anti-phosphorylated histone H2A.X at Ser^139^ (*γ*H2AX) antibodies in NB-39-nu cells treated at the indicated time points with 1 *μ*M CHK1i; and (c) detection of S phase nuclei. NB-39-nu cells were treated with or without 1 *μ*M CHK1i for indicated time periods and then exposed to EdU for 20 min. One hundred nuclei stained with 4′,6-diamidino-2-phenylindole (DAPI, nuclear DNA, blue) were counted in each of the triplicate sets and the relative numbers of EdU-labeled S phase nuclei (red) were scored. Data are presented as the mean ± SD. ^*∗*^*P* < 0.05 and ^*∗∗*^*P* < 0.001.

Recently, we reported that CHK1i (PF-477736) induced p53 activation and its downstream transcriptional targets such as p21 and MDM2 in the NB-39-nu neuroblastoma cell line which was less sensitive to CHK1i [5]. To assess the increased expression of MDM2 mRNA in response to CHK1i, we performed RT-qPCR analysis in seven neuroblastoma cell lines: NB-39-nu, SMS-SAN, NBLS, CHP134, SH-SY5Y, SK-N-AS, and SK-N-BE treated with 1 and 5 *μ*M PF-477736 for 24 hours or without PF-477736. We found that MDM2 mRNA levels were elevated by the treatment of PF-477736 in most of the cell lines except SK-N-AS which has a homozygous deletion of the p53 gene locus [17] (Figure 1(a)).

Further, we assessed whether MDM2 protein could be increased in response to CHK1i. For this purpose, a time-course experiment was performed in NB-39-nu cells treated with 1 *μ*M PF-477736 for 10 minutes up to 300 minutes and without PF-477736. Remarkably, MDM2 protein was transiently increased at time points prior to DNA damage response as judged by detection of the histone variant phospho-histone H2A.X at Ser139 (*γ*H2AX), which are markers for double-strand breaks and the phosphorylated form of p53 at Ser-15. In addition, an increase of p21 protein was observed in parallel with that of p53 phosphorylation (Figure 1(b)).

Previous studies reported that CHK1i exposure resulted in catastrophic DNA damage in the S phase of the cell cycle and decreased DNA replication capacity [18–20]. We, then, conducted S phase nuclei staining to investigate whether replication defects could be observed in the initial response to PF-477736 in NB-39-nu cells. For this purpose, NB-39-nu cells were treated with or without 1 *μ*M PF-477736 for 1 hour or 2 hours and then exposed to EdU for detecting S phase nuclei. As shown in Figure 1(c), the EdU uptake was significantly attenuated within 1 h and 2 h after treatment with PF-477736 (*P* < 0.05 and *P* < 0.01, resp.). These results suggested that CHK1i-induced replication defects appeared as an earlier response than the DNA damage response and might occur in parallel with elevated levels of MDM2 protein.

### 3.2. CEP131 Interacts with MDM2 in Response to CHK1i

**Figure 2:**
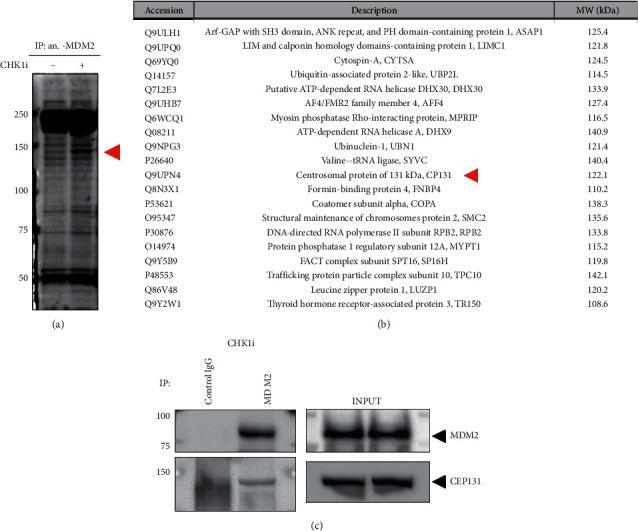
Centrosome-associated protein 131 (CEP131) was identified as an MDM2 interacting protein in response to CHK1i (PF-477736). (a) Coomassie staining of anti- MDM2 immunoprecipitates. Whole-cell extracts from NB-39-nu cells treated with 1 *μ*M CHK1i or DMSO (NT) for 24 h were immunoprecipitated (IP) with anti-MDM2 antibody. The densely stained band ranging in molecular weight from 100 to 150 KDa (Red arrow) was recovered and analyzed by mass spectrometry. (b) Mass spectrometric analysis. The listed proteins were shown in descending order of peptide coverage. The red arrow indicates CEP131. (c) Immunoprecipitation analysis with MDM2 and CEP131. NB-39-nu cells were treated with 1 *μ*M CHK1i for 24 h and were coimmunoprecipitated (IP) with mouse control IgG or with anti-MDM2 antibody. The immunocomplexes were resolved and immunoblotted with the indicated antibody. The immunoblots using whole-cell lysates ensured equal loading (INPUT).

To explore the functional role of MDM2 in the initial response to CHK1i, we conducted affinity purification and mass spectrometry to identify protein complexes in MDM2 immunoprecipitates. For this reason, NB-39-nu cells were treated with or without 1 *μ*M PF-477736 for 24 h and immunoprecipitated with anti- MDM2 antibody. The immunocomplexes were separated by electrophoresis and stained to visualize as band staining. As shown in Figure 2(a), we detected a significant densely stained band between 100 and 150 kDa and obtained a series of candidate interacting proteins with MDM2 in response to PF-477736 (Figure 2(b)). Among them, centrosome-associated protein 131 (CEP131), which has been reported to be associated with several types of cancer, was further investigated. We then confirmed the interaction between MDM2 and CEP131 by a coimmunoprecipitation experiment. NB-39-nu cell was treated with 1 *μ*M PF-477736. Twenty-four hours after the treatment, the whole-cell lysates immunoprecipitated with anti-MDM2 antibody or control IgG followed by anti-CEP131 antibody. As shown in Figure 2(c), CEP131 was detected in the MDM2 immunoprecipitate prepared from NB-39-nu cells treated with PF-477736. These results indicate that CEP131 is a newly identified interacting partner of MDM2 in response to CHK1i.

### 3.3. CEP131 Has a Prognostic Impact and Is Negatively Regulated by MDM2 in Neuroblastoma

**Figure 3:**
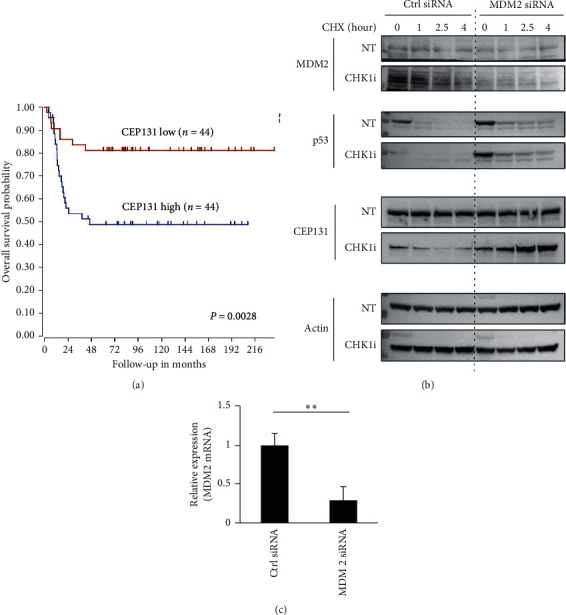
CEP131 is correlated with unfavorable prognosis of neuroblastoma and is negatively regulated by MDM2. (a) Kaplan–Meier survival curves of a cohort of 88 neuroblastoma patients stratified by high or low CEP131 mRNA expression. (b) Protein stability assays: NB-39-nu cells were transfected with MDM2 siRNA or negative control siRNA. Forty-eight hours after transfection, cells were treated with 1 *μ*M PF-477736 (CHK1i) or DMSO (NT) for 30 min and then cycloheximide (CHX) was added for indicated time periods. (c) Quantitative RT-PCR analysis of MDM2 mRNA levels in NB-39-nu cells transfected with control siRNA (ctrl siRNA) or MDM2 siRNA. ^*∗∗*^*P* < 0.01.

We asked whether CEP131 expression could be associated with prognosis in patients with neuroblastoma. We analyzed CEP131 mRNA levels based on the publishing data set (Tumor Neuroblastoma public-Versteeg-88-MAS5.0-u133p2) by R2, a Genomics Analysis and Visualization Platform, and found elevated CEP131 expression in neuroblastoma patients (*n* = 88) that correlated with an unfavorable prognosis (*P*=0.0028, Figure 3(a)).

To clarify a possible functional relationship between MDM2 and CEP131, we determined whether MDM2 exhibits ubiquitin ligase activity on CEP131. However, an *in vitro* assay revealed that CEP131 was not a substrate for MDM2 ubiquitination activity (data not shown). To assess the possible indirect influence on protein stability of CEP131 by MDM2 in response to CHK1i, we performed cycloheximide (CHX) chase assay in NB-39-nu cells with siRNA-mediated knockdown of MDM2 and compared it with a control. Interestingly, when protein synthesis was blocked by CHX, we observed that, in contrast to MDM2 stabilization, CEP131 protein was destabilized following exposure of cells to PF-477736 (Figure 3(b), left). Furthermore, siRNA-mediated depletion of MDM2 resulted in a reversion back to CEP131 stabilization despite the presence of PF-477736 (Figure 3(b), right). Consistent with the previous reports showing that MDM2 exerts ubiquitin ligase activity towards p53 [21], we found that siRNA-mediated knockdown of MDM2 stabilized p53 protein in the presence or absence of PF-477736 (Figure 3(b), right). These results indicated that MDM2 was required for CEP131 protein destabilization following the exposure of cells to CHK1i. As shown in Figure 3(c), we confirmed that the knockdown efficiency of MDM2 was sufficient for the experiment in mRNA levels.

Collectively, CEP131 may have prognostic potential for unfavorable neuroblastoma patients and be negatively regulated by MDM2 in neuroblastoma.

### 3.4. CEP131 Abrogates CHK1i-Induced Replication Stress

**Figure 4:**
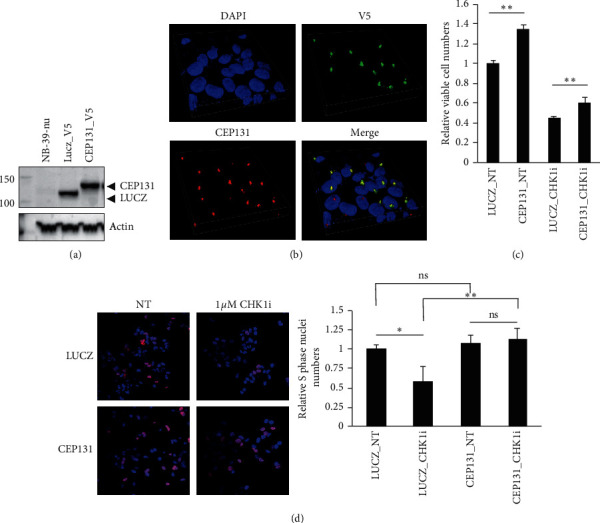
CEP131 promotes cell growth and rescues replication defects in NB-39-nu cells. (a) Western blotting for NB-39-nu cells stably integrated V5-tag-conjugated CEP131 gene (CEP131-V5tag), V5-tag-conjugated LUCZ gene (LUCZ-V5tag), or No lentiviral transduced cells (NB-39-nu). (b) Colocalization of endogenous and exogenous CEP131 was detected by immunofluorescence staining analysis (yellow). The lentiviral-mediated exogenous CEP131 was detected with anti-V5 antibody (green). Both endogenous and exogenous CEP131 proteins were detected with anti-CEP131 specific antibody (red). (c) AlamarBlue cell viability assay. LUCZ-V5tag cells (LUCZ) and CEP131-V5tag cells (CEP131) were treated with 1 *μ*M PF-477736 (_CHK1i) or DMSO (_NT) for 48 h and analyzed for cell viability. Data are presented as the mean ± SD of triplicates. (d) Detection of S phase nuclei. CEP131-V5tag cells and LUC-V5tag cells were treated with 1 *μ*M PF-477736 (CHK1i) or DMSO (NT) for 30 min and then exposed EdU for 10 min. S phase nuclei (red) were scored. ^*∗*^*P* < 0.05. ^*∗∗*^*P* < 0.01. ns: not significant.

To study the biological function of CEP131 in response to CHK1i, we generated a NB-39-nu cell line with lentiviral-mediated stable expression of V5-tag-conjugated CEP131 gene and LUCZ (Figure 4(a)). We confirmed that the exogenously introduced-CEP131 proteins were properly localized with endogenous CEP131 (Figure 4(b)). To examine the effect of CEP131 on neuroblastoma cell growth, a cell viability assay was performed. As expected, the CEP131-transduced cells exhibited increase in cell viability as compared with the LUCZ-transduced cells (Figure 4(c), LUCZ_NT versus CEP131_NT, *P* < 0.01). Notably, the predominant effect on cell viability by integrated CEP131 was retained in the presence of PF-477736 (Figure 4(c), LUCZ_CHK1i versus CEP131_CHK1i, *P* < 0.01). We further investigated whether integrated expression of CEP131 could affect DNA replication in the presence or absence of CHK1i. As shown in Figure 4(d), EdU-uptake was significantly increased in CEP131-transduced cells compared with LUCZ-transduced cells in the presence of PF-477736 (Figure 4(d), LUCZ_CHK1i versus CEP131_CHK1i, *P* < 0.01), whereas no remarkable difference was observed in absence of PF-477736 (Figure 4(d), LUCZ_NT versus CEP131_NT, *P* > 0.05). Interestingly, we observed no significant difference between the presence and absence of PF-477736 in CEP131-transduced cells (Figure 4(d), CEP131_NT versus CEP131_CHK1i, *P* > 0.05). Collectively, these results suggested that overexpression of CEP131 might have a protective role in response to CHK1i-induced replication stress.

## 4. Discussion

In this study, we observed that CHK1i induced transient increase of MDM2 protein expression in neuroblastoma cells. Further, MDM2 interacts with CEP131 and may be causally associated with CEP131 protein degradation. However, in our experimental condition, CEP131 is not likely to be a direct target of ubiquitination by MDM2. Since it has been reported that E3 ubiquitin ligase MIB1 directly targeted CEP131 as ubiquitination substrate [22], MDM2 might affect this interaction through an unknown mechanism. Then, we newly found that high expression of CEP131 was correlated with unfavorable prognosis of neuroblastoma patients and accelerated cell proliferation in neuroblastoma. Supporting our notion, CEP131 has been recently recognized as an important cancer-related gene in several human malignancies [14–16, 23, 24]. However, a direct link between CEP131 function and cancer promotion remains unclear.

Recently, two independent groups provided mechanistic insight into CEP131, which regulates centrosome duplication [24, 25]. Although the identifying phosphorylation sites on CEP131 were different in the two groups, they reported that CEP131 was phosphorylated by Polo-like kinase 4 (Plk4) to form proper centriolar satellite organization. Furthermore, overexpression in colon cancer HCT116 cells accelerated tumor formation and growth in nude mice [24], and this was consistent with the findings of a previous study showing that tumor growth was significantly suppressed in athymic mice transplanted breast cancer MCF-7 cells in which CEP131 expression was depleted [14]. Taken together, these observations indicate that the increased expression of CEP131 can result in disrupting proper centrosome duplication leading to aneuploidy and chromosomal instability [14, 24, 26]; activating proliferative pathway such as ERK and AKT signaling [15, 16]; inducing primary cilium [22, 27] and subsequent activation of Sonic Hedgehog pathway [23]; disrupting tissue architecture caused by centrosome aberrations leading to dissemination of potentially metastatic cells [28].

In the current study, we highlighted the possible role of CEP131 on replication stress (RS) in MYCN-amplified neuroblastoma NB-39-nu cell line. Increased levels of oncogene-induced RS are considered as a hallmark of neuroblastoma with MYCN amplification, a prominent predictive marker of unfavorable prognosis [29–32]. MYCN-mediated hyperproliferation signals increase the level of RS stress in neuroblastoma cells, thus rendering them reliant on CHK1 function to ensure their acceptable genome duplication cycles. Consequently, cells may become sensitive to CHK1i [33, 34]. Excessive RS-mediated cell death induced by either cytotoxic drugs or inhibitors, including the CHK1i, is known as replication catastrophe, suggesting a potential therapeutic window for targeting oncogene-induced RS [20]. With respect to RS, a previous study showed that UV or UV-mimetic agents induced CEP131 sequestration and blocked centriolar satellite formation, leading to primary cilium formation [20, 22]. As UV light induces RS such as DNA single-strand brakes, it is presumed that cancer cells overexpressing CEP131 may tolerate endogenous and/or exogenous replicative stress. This may facilitate their survival even in an oncogene-induced hyperproliferative state. On the other hand, it has been reported that MDM2 has an ability to induce RS by increasing unscheduled origin firing and accelerating S-phase entry [35], thereby partially supporting our interpretation that MDM2-mediated CEP131 degradation might result in increased RS. However, concerning accelerated S-phase entry by MDM2, it is still controversial because centrosome duplication, which is supposed to be related to CEP131 function, is initiated at G_1_/S transition. Although accumulating evidence indicates that centrosomes might be important for initiating S phase concurrent with DNA replication [26], a direct link between CEP131 function and DNA replication machinery remains largely unclear.

In our observation, although 30 min of short-term CHK1i treatment did not influence the numbers of S phase nuclei in CEP131-transduced NB-39-nu cells (Figure 4(a), CEP131_NT versus CEP131_CHK1i), 48 h of long-term CHK1i treatment clearly inhibited cell growth (Figure 4(c), CEP131_NT versus CEP131_CHK1i). This conflicting result indicated that CHK1i not only might induce RS but may also play an inhibiting role in cell proliferation by targeting the other phases of cell cycle such as G2/M transition. However, our present results suggested that unfavorable neuroblastomas might rely, in part, on CEP131 function to maintain their genome integrity against oncogene-induced RS in a hyperproliferative state. Further studies about the precise mechanism by which CEP131 protects cells from RS could open the window to discover therapeutic strategy targeting to CEP131 and/or other centriolar satellites for effective neuroblastoma treatment.

## Data Availability

The data used to support the findings of this study are available from the corresponding author upon request.

## Abbreviations

CEP131: Centrosome-associated protein 131
CHK1: Checkpoint kinase 1
CHK1i: CHK1 inhibitor
RS: Replication stress
SD: Standard deviation
CHX: Cycloheximide.

## Supplementary Materials

Supplementary Materials The materials and methods used in the study are given in this section.
