# Supplementary material for: CEP131 Abrogates CHK1 Inhibitor-Induced Replication Defects and Is Associated with Unfavorable Outcome in Neuroblastoma
Source: J Oncol. 2020 Sep 15;2020:2752417. doi: 10.1155/2020/2752417 (PMC7512061; doi:10.1155/2020/2752417)
Supplement: Supplementary Materials — The materials and methods used in the study are given in this section. [file 2752417.f1.pdf]

Supplementary Materials and Methods

Immunoblotting

Whole cell lysates were prepared in 1% NP-40 cell lysis buffer (Boston BioProducts, Ashland, MA, USA) or RIPA buffer (Nacalai Tesque, Kyoto, Japan) with cOmplete Mini Protease Inhibitor Cocktail (Roche, Basel, Switzerland) and phosphatase inhibitor cocktail (PhosSTOP, Sigma-Aldrich, St. Louis, MO, USA). Whole cell extracts (50–100 μg protein) were electrophoresed using an XCell SureLock Mini-Cell with 4-12% Bis-Tris gels or 3-8% Tris-acetate gels in MOPS, MES, or Tris-acetate running buffer (Invitrogen), respectively. Samples were transferred to nitrocellulose membranes (Bio-Rad, Hercules, CA, USA) and blocked with 5% bovine serum albumin (BSA) in Tris-buffered saline with 0.1% Tween (TBST). Membranes were then probed for overnight at 4°C with the following primary antibodies diluted in TBST/5% BSA: monoclonal anti-p53 (DO-1, Santa Cruz Biotechnology; 1:500), monoclonal anti-β-actin (AC-74, Sigma; 1:4000), monoclonal anti-MDM2 (SMP14, Santa Cruz Biotechnology, Dallas, TX, USA; 1:200), monoclonal anti-p21 Waf1/Cip1 (12D1, Cell Signaling Technology, Danvers, MA, USA; 1:1000), monoclonal anti-V5 tag (Invitrogen; 1:1000), polyclonal anti-p-p53-Ser15, polyclonal anti-p-Histone H2A.X-Ser139 (Cell Signaling Technology; 1:1000) or polyclonal anti-AZI1 (ab99379, Abcam, Cambridge, UK; 1:1000) antibody. Membranes were washed in TBST and probed with horse radish peroxidase (HRP)-coupled anti-rabbit or anti-mouse secondary antibodies (Cell Signaling Technology) diluted 1:2500 in TBST/5%

ECL Blocking Agent (GE Healthcare, Chicago, IL, USA) for 1 h. Signal intensities were detected by using a ChemiDoc XRS+ imaging system (GE Healthcare). The immunoblot of actin was performed as a loading control.

Immunofluorescence staining

LUCZ and CEP131 transduced NB-39-nu cells were grown on coverslips, fixed with 3.7% formaldehyde in PBS for 15 min, washed twice with PBS, and then permeabilized with PBS/0.2% Triton X-100 for 10 min. Cells were then washed twice with PBS/0.01% Triton X-100, blocked with PBS/3% BSA/0.01% Triton X-100 for 1 h, and then incubated with the indicated primary antibodies diluted in TBST/5% BSA for overnight at 4°C in a humidified chamber. Thereafter, cells were washed twice with PBS/0.01% Triton X-100 and incubated with Alexa Fluor 488-conjugated goat anti-mouse IgG (Molecular Probes, Eugene, OR, USA) and Alexa Fluor 568-conjugated goat anti-rabbit IgG (Molecular Probes) of the secondary antibodies at 1:500 in PBS/1% BSA/0.01% Triton X-100. Cells were mounted with Vectashield Mounting Medium containing DAPI (Vector Laboratories).
